# Counselling and psychotherapy post‐COVID‐19

**DOI:** 10.1002/capr.12325

**Published:** 2020-06-03

**Authors:** Panos Vostanis, Chance A. Bell

**Affiliations:** ^1^ Department of Neurosciences, Psychology and Behaviour University of Leicester Leicester UK; ^2^ Department of Family Science University of Nebraska Kearney Kearney NE USA

**Keywords:** counselling, psychotherapy, mental health, COVID‐19

## Abstract

We consider how the prolonged, complex and uncertain aftermath of the COVID‐19 crisis will present challenges and opportunities for counselling and psychotherapy. Increased mental strain on populations, individuals and professionals is likely to be compounded by further constraints in therapeutic resources. Nevertheless, emerging needs and priorities will offer ground for systems thinking in linking the application of a range of therapeutic frameworks, theories to address global challenges, integration of counselling and psychotherapy into new sectors, service models for the most vulnerable, use of digital approaches, support mechanisms for professionals and interdisciplinary research.

## 1 CHALLENGES TO OUR WELL‐BEING COVID‐19 CREATED

The world has entered unchartered waters that only time and history will make sense of. We may not even be at the beginning of a chain of events and changes that will define at least the next generation. This is no excuse for not attempting to anticipate the impact on each one's field, or to articulate how we should respond in likely scenarios. So, what does this new era hold for counselling and psychotherapy? Crucially, where should counselling and psychotherapy position themselves to respond to evolving population needs?

War conflicts have been the closest parallels of global or regional turmoil. Even such periods of destruction and loss can be predicted to a certain extent, as causes are overt, gradual and externalised. The COVID‐19 crisis took world leaders, economic markets, healthcare systems and societies by surprise in its rapid escalation and impact on every single aspect of life. Its aftermath is speculative and obscure. Nevertheless, some previous lessons and evidence could be of value. The initial reduced suicide rates during World War II were attributed to populations fighting for survival, gaining a sense of purpose, and being in need of social integration (Lester & Yang, 1992). Psychological rebuilding and economic rebuilding were marked by a deep sense of not only loss and inequalities, but also aspirations and hope for the future. To a lesser extent, similar lessons could be learnt from the SARS pandemic that led to both maladaptive and empathic strategies (Puterman, Delongis, Lee‐Baggley, & Greengrass, 2009). A common tragedy thus has the potential to unify us in moving forward, challenging previous systems and theories, and fertilising ideas and collaborations, nationally and globally.

In our case, causes and effects appear inextricably intertwined. We face sudden changes in lifestyle, self‐isolation that transgresses socioeconomic strata, fears of health and economic contagion, uncertainty on the duration and outcome of the crisis, and visible collapse of economic industries that go beyond individual job losses (World Health Organization, 2020a). Social media serve both our interconnectedness with family, friends and colleagues, and a less welcome 24‐hr reinforcement of our anxieties. Crucially, no one is immune. Different age groups face somewhat differential threats. For many of the young, at least in relatively stable societies and communities, this is a collapse of their known safety and stability. Adults are torn between individual, family and occupational risks. The elderly bear disproportionate fatality rates and are ‘punished’ with additional measures of isolation, albeit for their own protection. Socioeconomic factors and social issues pose additional risks to ethnic minority and disadvantaged communities, as well as to vulnerable groups, such as victims of domestic violence.

Consequently, it is a reasonable prediction that the rates of a range of mental health conditions and relational tensions will steadily increase, with the previously described risk factors exerting a cumulative effect over time. Acute and prolonged presentations include panic attacks, generalised anxiety, post‐traumatic stress symptoms and depression (Qiu et al., 2020). Prevalence rates of post‐traumatic stress symptoms are likely to increase sharply among healthcare workers. Women and children are particularly vulnerable as they face increased abuse risk (Bradbury‐Jones & Isham, 2020). Disruption of therapies, service provision and social supports will result in exacerbation or recurrence of existing mental health problems. Social anxiety is a likely outcome of self‐isolation, social distancing and various types of enforcement (Alden & Auyeung, 2014). Whatever the exit strategies of lockdown and their effectiveness, notwithstanding the risk of further peaks in COVID‐19 rates, psychological adjustment to previous social interactions may share features of societal reintegration following treatment in intensive care units and facing near‐death experiences (Papathanassoglou, 2010), or release from custodial or other residential settings (Semenza & Link, 2019).

It is plausible that individuals, groups and communities will go through a similar process of grief and mourning for lost ones to those experienced by health professionals (Kubler‐Ross, 1967; National Cancer Institute, 2011). Even this process will be out of the ordinary for so many who were unable to say goodbye to loved ones and attend their funerals. When people have mourned their losses, they will also seek answers on whether they were sufficiently protected by governments and responsible organisations, a type of anger that they may not be able to process because of the complex nature of the COVID‐19 crisis. Budget cuts are likely to spread into welfare and education, thus depriving further those in mental health need of supports and preventive measures on the interface between health and social care.

Prevalence rates of mental health problems usually increase in areas of disadvantage during times of difficulty, which also have the least access to therapeutic resources. Underlying risk factors such as domestic violence and alcohol abuse have been shown to increase during isolation (Bradbury‐Jones & Isham, 2020; World Health Organization, 2020b), while social distancing is extremely difficult in overcrowded communities, such as informal settlements in low‐income countries (Abuya, Austrian, Isaac, & Kangwana, 2020) and refugee camps (Vince, 2020).

The inverse correlation between the level of mental health needs and service provision for vulnerable groups is a long‐standing public health paradox and injustice. Those with the highest level of need, such as the homeless, have the least access to services (Vijayaraghavan et al., 2012). This service pattern is a barrier to counselling and psychotherapy for both preventive and intervention purposes. Despite the wider availability of evidence‐based therapeutic programmes, such resources have become increasingly stretched within the public sector in recent years (Scott, 2018), which COVID‐19 may only exacerbate. This uneven ratio between demand and supply will deteriorate dramatically in the COVID‐19 aftermath, as health and government budgets are drained by acute healthcare costs being diverted towards testing, equipment, intensive treatment and, hopefully, vaccination in the near future.

The resulting economic recession will increase all the previously stated risk factors and place further strain on resources (Barbaglia, ten Have, Dorsselaer, Alonso, & de Graaf, 2015). This will hit the youth, unskilled workers and communities already high on unemployment and criminality harder. However, in contrast to previous and relatively short‐term periods of recession, there may well be a qualitative difference in the next economic cycle, at least in the short to medium term (McKibbin & Fernando, 2020). Previously stable and rising industries such as tourism, entertainment, travel and retail will be hit hard and exert a knock‐on effect through a globalised supply chain on a range of producers in low‐income countries and providers in more affluent economies (Maital & Barzani, 2020). Consequently, the mental strain may not only be felt in large numbers of individuals in search of employment, but could also spiral to those in insecure posts, companies or industries. In spite of the substantially greater level of need, the predicted recession will hinder commissioners from diverting funding to already‐stretched mental health services. Consequently, the existing disparity between physical and mental health budgets may well widen further.

## 2 COUNSELLING AND PSYCHOTHERAPY MOVING FORWARD

Although counsellors and therapists will not be exempt from such knock‐on effects, challenges will also open new ways of moving forward in their profession by responding to individual and societal needs, as discussed in the following sections. For example, they will have been exposed to similar health risks, isolation, grief and economic uncertainty, individually and with their families. Therapists can join clients around these common themes, harnessing their own experience to further therapeutic progress (Aponte & Ingram, 2018). In an earlier study in a war‐affected region, we found that the higher rates of post‐traumatic stress and other mental health presentations among frontline healthcare professionals were predicted by their personal traumatisation through direct and indirect exposure to conflict and loss, rather than through professional exposure to trauma (Shamia, Thabet, & Vostanis, 2015). Professional insecurity is bound to follow, whether working in the public or private sector.

## 3 THEORETICAL USE AND RE‐EXAMINATION

As we delve into the unknown, we cannot claim to have prescriptive solutions. However, we have the responsibility to seek a direction and debate for the field. We have considerable tools to draw from theory, practice and evidence, but we also need to connect and apply those to new circumstances, as well as to develop new ones. This wealth of different theoretical approaches offers many lenses to help us understand and make sense of the impact of the current situation. One reason for which key counselling and psychotherapy theories continue to influence practice is that their frameworks and objectives adapt to different socioeconomic and intergenerational contexts. We may thus need to draw on them while trying to experience and comprehend the COVID‐19 aftermath, which is by no means an easy task, and consider new ways of integrating them.

Drawing some parallels with the use of key modalities with individual clients or client groups could be a starting point. We fortunately have access to a range of trauma reprocessing interventions, and their principles of helping clients make sense of different kinds of abuse and lesser types of trauma that, nevertheless, impact their lives (Marzillier, 2014). To replicate this approach at a collective level, we should aim for a conceptual understanding of what led to the COVID‐19 crisis and its potential consequences, as we can no longer claim that there will be no further crises of similar or different (e.g. ecological) kinds. Humanistic approaches to counselling (Rogers, 1951) may need to challenge why values were lost and how they could re‐emerge.

Attachment‐focused modalities (Hughes, 2017) could help us tackle our own attachment representations of which relationships really matter and how to regain their security, while confronting our inner existential conflicts (Frankl, 1946) is more timely than ever, albeit in conjunction with external challenges. Long ignored or denied existential questions may emerge from the recesses of the human psyche seeking greater understanding than in many generations, for example queries such as ‘What matters most in life?’‘What is the purpose of this life?’ ‘Is there life after death?’ and ‘Why do people suffer?’ Counsellors should expect such inquiries to arise with clients and from within themselves. We would do well to lean into the meaning‐making frameworks of those we see. The often‐taboo topic of spirituality and religion may well merit extra attention, and even demand it during a time of global challenge. Because of this, perhaps now, the age‐old meaning‐making traditions can shake off the scientific and academic chains which bind them and merge with them to guide those searching for direction and seeking hope.

Expanding mindfulness‐based programmes are informed by spiritual sources that originated centuries ago, and are pertinent in strengthening our inner resilience, while lessening dependencies on frail comforts and external factors that may remain beyond our control (Woelde, Hechanova, Ramos, Macia, & Moschetto, 2018). The repositioning of counselling and psychotherapy in response to societal needs will benefit from a systemic understanding (Dallos & Vetere, 2009) of how families, communities, services, professional groups and societies evolve locally, nationally and globally. Nevertheless, we will need to question whether our theoretical inheritance is enough to move us forward, and whether we should be developing theories that connect psychological functions and mechanisms with the prominent challenges of our time.

We can equally draw on frameworks to inform how counselling and psychotherapy should respond to changing population needs. Hanisch’s (1970) stance that the ‘personal is political’ already has several implications for practice in relation to social justice (Winter, 2019) and power dynamics (Proctor, 2017). Socioecological systems theory (Bronfenbrenner, 1979) extends these implications by placing the client–therapist relationship within dynamically linked systems of family, community and society (including culture, religion, beliefs and services). If the estimates of economic recession and widening socioeconomic gaps are approximately accurate, increasing mental health needs can only be addressed if counselling and psychotherapy are positioned within an overall health and welfare policy and service model. There is strong evidence that the higher the level of disadvantage, the lower the availability of and access to needs‐led therapeutic help (Waldegrave, 2005). Although counselling and psychotherapy have been a low priority in gradually weakened public health systems, some positive signs are emerging from the COVID‐19 crisis in public, media and political support for public health care and professionals, and on decisions being informed by scientific evidence. As the role of public health had to be re‐invented and strengthened because of, rather than in anticipation of, the crisis, it may prove difficult and politically unpopular for governments to reverse this trend.

The importance of sustaining the mental well‐being of healthcare professionals and society has not escaped policy drivers either (World Health Organization, 2020a). What should be articulated more clearly is the integration of counselling and psychotherapy in health and welfare services, in order to better address population needs and to avoid commissioning economic dilemmas. To achieve this, practice needs to be reframed and interventions to be redesigned before they are implemented in settings of disadvantage. Within a comprehensive scaled service model, a ‘frontline’ response is not necessarily mutually exclusive with second‐level response and longer‐term therapies. Focused programmes could be complemented by consultation and training to other agencies. The use of digital applications of practice, supervision, consultation and training has been enhanced during self‐isolation, so has our confidence in using them (Greenhalgh, Wherton, Shaw, & Morrison, 2020; Liu et al., 2020). Building on these strengths will make counselling and psychotherapy skills both more accessible and valued (including between high‐ and low‐income countries).

A paradoxically similar adaptation could be required within the private sector. Although coaching, occupational and sports psychology are already widely established, albeit with ad hoc counselling input (Grant & Green, 2018), employers may view staff well‐being (indeed their own) in a new light. Retention and productivity in the face of a shrinking economy, increasing market competition and workforce mental ill health should lead to integrated counselling structures that are not dissimilar to the expanding student counselling services within Universities (Barkham et al., 2019). Such therapeutic support, which goes beyond pastoral care, is viewed by an increasing number of academic institutions as essential in attracting undergraduate and postgraduate students. This, however, will be no mean task. With industry budgets under pressure, professional and research bodies should provide convincing arguments and supporting evidence that such services will deliver demonstrable results and cost savings.

Support for other disciplines and agencies, changes in roles and models, and exposure to their own employment stressors and uncertainties for counsellors and psychotherapists will require transformation in training programmes and curricula, supervision and support such as providing experiential opportunities and ongoing career advice on the adaptation of professional roles. Professional associations will have new opportunities and responsibilities in addressing these emerging needs and challenges and in setting the vision and strategy for the professions. In order to respond to societal changes, professional bodies will need to interact more with services, employment and client stakeholder groups. Such collaborative partnerships should strive to achieve greater access to counselling and psychotherapy, adapted to the specific requirements of those in the highest need. These collaborations can in turn influence local and national policy and guidelines. Parallel integration will be essential for academic and other training providers. Systems thinking (‘we cannot act alone’) is thus likely to permeate and link all levels related to counselling and psychotherapy.

New needs, opportunities and ways of thinking should both drive and be driven by evidence. Research should be a core component of all previously discussed strategic directions. Programme design and evaluation should be conceptually driven, methodologically robust and contextualised, for example by being informed by approaches such as realist evaluation (Pawson & Tilley, 1997) and science implementation (Blasé, van Dyke, & Fixsen, 2013). If counselling and psychotherapy are to be adapted and tested to meet population needs, so should their underpinning research. Disciplines such as international politics, economics, business management, anthropology and geography can cross‐fertilise new and complex ideas, and thus lead to findings and dissemination that truly reflect a rapidly changing world. As interdisciplinary researchers are not likely to come together without defined common objectives and criteria, this opens another leadership opportunity for the profession and its academic field to make a difference.

## 4 HOPE

Last but not least, individuals and families will need to make sense of the surprising overwhelm COVID‐19 caused to societies, healthcare systems, economies and governments. Individuals and families face crises regularly (e.g. death of a loved one, job loss and violence), and struggle and difficulty are part of the human condition. While the crisis caused by COVID‐19 surely has troubling features, as previously noted, a unifying opportunity simultaneously appears, with counselling and psychotherapy well positioned to champion the process. Counsellors, by virtue of the profession, are called upon to instil hope in clients. Individuals seek psychotherapy because they believe the therapeutic process and experience can provide relief from their suffering. This belief, this hope, rests within each counsellor and forms a vital component of effective treatment (Duncan, Hubble, & Miller, 1995). Thus, even more so at this time, clients will come seeking hope to navigate the aftermath of COVID‐19, and answer difficult questions.

In conclusion, mental health strain and needs are likely to increase in a variety of forms in the wake of COVID‐19. Clients and therapists alike do now and will continue to face significant challenges, providing a common theme for them to join around. New and altered welfare and social service policies are likely to emerge, thus impacting the allocation of funding for such, with the unfortunately high probability of excluding those who need it most. Despite the challenges COVID‐19 will cause, it provides opportunity for counsellors and therapists to lean into current theoretical frameworks, and test their applicability, while supplying fertile ground for theory development, and collaboration across disciplines. Training programmes and organisations will need to revise their current operational methods to ensure individual professionals and the mental health field are prepared to adapt and face future challenges, which surely will come. As we move through this together, we must answer the call to counter despair and provide hope.
